# Alternative pathways for hydrogen sink originated from the ruminal fermentation of carbohydrates: Which microorganisms are involved in lowering methane emission?

**DOI:** 10.1186/s42523-021-00153-w

**Published:** 2022-01-06

**Authors:** Ana Margarida Pereira, Maria de Lurdes Nunes Enes Dapkevicius, Alfredo E. S. Borba

**Affiliations:** Faculdade de Ciências Agrárias e do Ambiente, Instituto de Investigação em Tecnologias Agrárias e do Ambiente (IITAA), Universidade dos Açores, Campus de Angra do Heroísmo, rua Capitão João d’Ávila, 9700-042 Açores Angra do Heroísmo, Portugal

**Keywords:** Acetogenesis, H_2_ sink, Methanogenesis, Microorganisms, Propionate, Rumen fermentation

## Abstract

Agriculture is responsible for a great share of the anthropogenic sources of greenhouse gases that, by warming the earth, threaten its biodiversity. Among greenhouse gas emissions, enteric CH_4_ from livestock is an important target to slow down climate changes. The CH_4_ is originated from rumen fermentation and its concentration is affected by several factors, including genetics and nutrition. Ruminants have an extraordinary symbiosis with microorganisms (bacteria, fungi, and protozoa) that ferment otherwise indigestible carbohydrates, from which they obtain energy to grow and continue actively producing, among other products, volatile fatty acids, CO_2_ and H_2_. Detrimental ruminal accumulation of H_2_ is avoided by methanogenesis carried out by Archaea methanogens. Importantly, methanogenesis is not the only H_2_ sink pathway. In fact, other bacteria can reduce substrates using metabolic hydrogen formed during carbohydrate fermentation, namely propionate production and reductive acetogenesis, thus lowering the CH_4_ produced. Although the complexity of rumen poses challenges to mitigate CH_4_ production, the emergence of sequencing techniques that allow the study of microbial communities, gene expression, and metabolome are largely contributing to unravel pathways and key players in the rumen. Indeed, it is now recognized that in vivo emissions of CH_4_ are correlated to microbial communities, and particularly with the abundance of methanogens, several bacterial groups, and  their genes. The goal of CH_4_ mitigation is to work in favor of the natural processes, without compromising rumen function, animal health, and productivity. Notwithstanding, the major challenge continues to be the feasibility and affordability of the proposed solutions.

## Introduction

Global warming threatens biodiversity alongside the life of humans. Among other gases, the release of CO_2_ and CH_4_ into the atmosphere contributes significantly to the greenhouse effect, a phenomenon that prevents the reflection of solar energy back from the earth's surface, causing a rise in temperature [1]. Agriculture is responsible for a great share of the anthropogenic sources of greenhouse gases (GHG) [1]. According to FAO (2013), GHG emission from livestock represents 14% of human-induced emissions, being beef and dairy cattle the main contributors [2]. Within the livestock sector, feed production, processing, and transportation account for ≈ 45% of total GHG, followed by enteric CH_4_ emissions (≈ 40%) [2]. Therefore, acting over this latter source constitutes an opportunity to achieve the goals of the Green Deal, which seeks for a 55% cut in GHG emission by 2030, compared to 1990 levels [3].

**Fig. 1:**
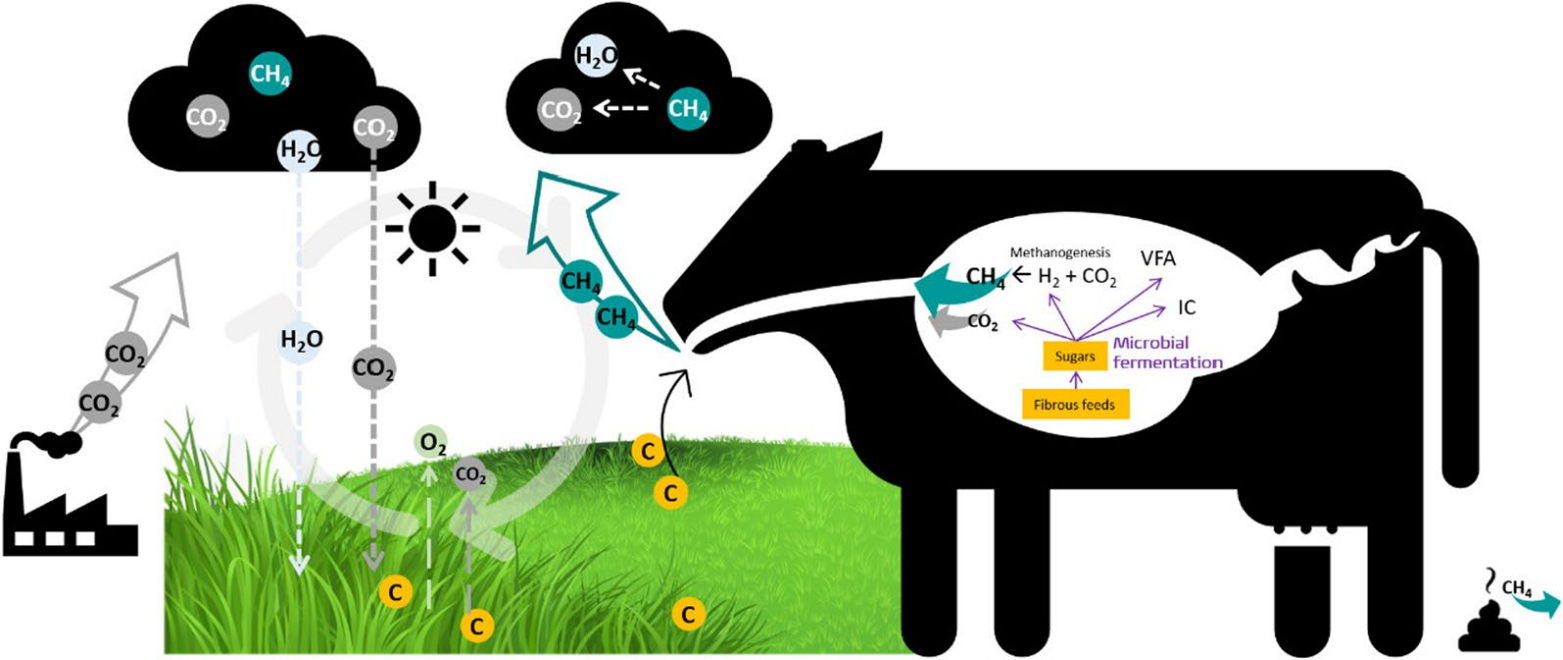
Simplified scheme of rumen methane production and emission and the biogenic carbon cycle. C designates carbon, fixated in plants from CO_2_ through photosynthesis, which is then consumed by animals as carbohydrates. VFA, volatile fatty acids; IC, intermediary compounds and/or other products

Although animal farming is often cited as the cause of the problem, it can actually be part of the solution. If other anthropogenic activities (e.g., burning of fossil fuels) slow down the emission of CO_2_, mitigation of GHG emission, or at least the share from the livestock sector, might be achieved by sequestration of carbon in grasslands [4]. Carbon from the atmosphere (CO_2_) is fixed in the soil mainly through plant photosynthesis and thus converted into organic material (e.g., grasses and forages), which is then consumed by grazing animals [5]. From the ruminal fermentation of carbohydrates, CH_4_ is produced at the expense of energy and released into the atmosphere, where after approximately 10 years, it is broken down and converted back into CO_2_, giving continuity to the natural carbon cycling, in a process called the biogenic carbon cycle (Fig. 1) [6]. Therefore, in theory, the reduction of animal production, which entails a decrease in protein availability, is not the only alternative to tackle GHG emission. Instead, enhancing animal productivity while decreasing CH_4_ might be a sustainable option, not compromising feed for a growing population. In the latest years, a lot of effort has been put into the study of animal breeding [7], vaccines [8], dietary management, and additives [9], as means to mitigate CH_4_ emissions. Despite positive results reported in some studies, there is still not a consensus at a global scale, mainly because the efficacy and feasibility of each proposed strategy are affected by several factors. Such include the farming system, acceptability of both consumers and farmers, policies, and financial support [10].

As the majority of CH_4_ is produced in the rumen, strategies for mitigating CH_4_ emissions, necessarily affect the rumen microbiome and vice-versa. The rumen is harbored by a consortium of microorganisms including protozoa, bacteria, archaea, and fungi that conjointly enable the fermentation of otherwise indigestible carbohydrates such as cellulose and hemicellulose into volatile fatty acids (VFA; e.g., acetate, propionate, and butyrate). Other products of carbohydrate fermentation include formate, ethanol, lactate, succinate, branched-chain volatile fatty acids, ammonia, CO_2_, and H_2_ [11]. Dissolved H_2_ and CO_2_ are utilized by methanogens, a group of microorganisms belonging to the Archaea domain, to form CH_4_. Despite being the main pathway to avoid the accumulation of H_2_ in the rumen, methanogenesis constitutes a loss of energy, reflected in animal productivity (e.g., methane emission has been correlated to residual feed intake [12]) and is strongly affected by diet (e.g., the level of concentrate has been correlated to CH_4_ yield in grazing cows [13, 14]). However, methanogenesis is not the only H_2_ sink mechanism in the rumen [15], being alternative pathways worth exploring. Therefore, the present work aims to summarize mitigation of CH_4_ production, via alternative H_2_ disposal pathways, namely, propionate production and reductive acetogenesis.

## Microbial composition of the rumen

The rumen is a foregut ecosystem that hosts an enormous number of microbes living in symbiosis with the host. It is estimated to harbor a concentration of archaea of 10^7^ to 10^9^ cells/ml, bacteria of 10^10^ to 10^11^ cells/ml, protozoa of 10^4^ to 10^6^ cells/ml, and fungi of 10^3^ to 10^6^ cells/ml [16]. Putting these into perspective, the number of ruminal microbial cells of an adult dairy cow (estimated volume of 50 to 200 L) [17] is 40 to 500 times the number of human cells constituting an adult body (assuming the estimation of 3.72 × 10^13^ total cells by Bianconi et al. [18]).

### Archaea

Archaea in the rumen consist of methanogens belonging to the phylum Euryarcheota [19]. It is represented by four orders Methanobacteriales, Methanococcales, Methanomicrobiales, and Methanosarcinales [20]. Methanobacteriales include the genera *Methanobacterium* and the dominant genus *Methanobrevibacter*, which is divided into two clades, (1) clade *Methanobrevibacter gottschalki* that also includes the species *Mbb. thaueri* and *Mbb. millerae;* and (2) clade *Methanobrevibacter ruminantium* that also includes *Mbb. olleyae* [21]. Most methanogens use H_2_ for the reduction of CO_2_ into CH_4_ [22], although formate might also be used instead of CO_2_ by M*ethanobrevibacter*, some strains of *Methanobacterium* spp., and Methanomicrobiales, such as genus *Methanomicrobium* [20]. In addition, methanogens of order Methanococcales and Methanosarcinales can utilize methyl groups (e.g., genus *Methanosphaera* also uses methanol, whereas genera *Methanosarcina* and *Methanimicrococcus* also use methylamines), and acetate (e.g., *Methanosarcina*) to produce CH_4_ [20]. Methanol is originated from the demethyoxylation of dietary pectins, whereas mono-, di-, and tri-methylamines are mainly end-products of plant phosphatidylcholine degradation [23]. The structure of the methanogenic community at the species or strain level has been correlated with feed efficiency [24]. Moreover, some authors argue that feed efficiency is related to CH_4_ emissions [12, 25], although the link between both is complex and influenced by multiple parameters related to the rumen microbiome [26] and host factors (e.g., passage rate and nutrient absorption) [27]. Importantly, dominant archaea groups were found similar in samples collected from ruminants across the globe, which is likely an advantage to develop and implement worldwide strategies to mitigate CH_4_ emissions targeting methanogens [19].

### Bacteria

The rumen harbors cellulolytic and non-cellulolytic bacteria, being the first able to degrade cellulose and hemicelluloses. Primary cellulose fermenters are *Fibrobacter succinogenes*, *Ruminococcus flavefaciens,* and *Ruminococcus albus*. These bacteria are non-motile, adhering extensively to the fibers through the glycocalyx, and having cellulases located on the cell surface [28]. They hydrolyze cellulose and other polysaccharides (e.g., hemicelluloses and pectin), producing cellodextrins to utilize as a source of energy and make available for cross-feeding [28]. This is important to provide nutrients for the growth of other bacteria and/or non-adherent cells of the same species that are poised to adhesion to new feed particles [29]. Secondary cellulose fermenters, including *Butyrivibrio fibrisolvens*, *Clostridiurn longisporum*, and *Clostridium locheadii*, might be motile or non-motile, adhering minimally to fibers, and having extracellular cellulases [28]. Non-cellulolytic bacteria, able to degrade starch, hemicelluloses, or pectin, might include *Prevotella ruminantium*, *Eubacterium xylanophilum, Ruminobacter amylophilus*, *Succinimonas amylolytica*, *Succinivibrio dextrinosolvens*, *Selenomonas ruminantium*, *Selenomonas lactilytica*, *Lachnospira multiparus*, *Streptococcus bovis*, and *Megasphaera elsdenii* [30].

Despite the great diversity of bacterial species in the rumen, an extensive study reported the existence of 30 most abundant bacterial groups comprising ≈ 89% of total sequences found on livestock species [19]. Among them, *Prevotella*, *Butyrivibrio*, and *Ruminococcus*, unclassified *Lachnospiraceae*, *Ruminococcaceae*, Bacteroidales, and Clostridiales were predominant, having an abundance of ≈ 67%. Later works analyzing metagenome-assembled genomes (MAGs) revealed the existence of new genomes from the Actinobacteria, Fibrobacteres, and Proteobacteria phyla, also highlighting the abundance of genus *Succinivibrio* [31].

The existence of a core ruminal bacterial microbiome has been suggested, despite a clear variation associated with host and diet [19]. Indeed, a study reported that the breed is more determinant for metabolites and bacterial communities of bovines than diet and life-stage [32]. Even though the age and time of weaning significantly affect the diversity and abundance of rumen microbial communities [33], it has been suggested that colonization of rumen starts in utero, as samples collected in goat fetuses allowed the identification of sequences mainly belonging to the phylum Proteobacteria [34]. Also, three moments were shown to produce important shifts in rumen bacteria: delivery, milk intake, and weaning [34]. Moreover, it was reported that the rumen microbial colonization is affected differently by natural or artificial milk feeding systems [35], and the inoculation of young ruminants with fresh rumen fluid from adult animals enhanced microbial colonization, likely improving rumen development [36]. Furthermore, early life modulation of the rumen microbiome has been a matter of study regarding its effectiveness when compared to later interventions for the improvement of rumen fermentation and reduction of CH_4_ emission [37].

### Protozoa

Contrary to archaea and bacteria, ciliate protozoa vary across ruminants of different species as well as individuals of the same species [19]. A study in cattle indicated that *Entodinium*, *Diplodinium*, *Eremoplastron*, *Ostracodinium*, *Eodinium*, *Epidinium*, *Isotricha*, and *Dasytricha* were among the most abundant genera (> 1%) of a total of 13 identified in the ruminal fluid by microscopic identification and counting [38]. Conversely, in sheep and goats, 12 and 8 genera were identified, being *Dasytricha*, *Entodinium*, *Eudiplodinum*, *Diplodinium*, *Isotricha*, and *Metadinium* the more abundant (> 1%) in both species, and *Enoploplastron*, *Ophryoscolex*, and *Polyplastron* only in sheep [39].

Protozoa attach to the surface of partially digested feed particles, in which other microorganisms exert high fibrolytic activity, allowing them to take advantage of monosaccharides (e.g., glucose, cellobiose, and cellodextrins) that they use as their source of energy for growth and metabolism [40]. Also, protozoa predate bacteria from which they obtain amino acids for growth and maintenance [41]. Non-surprisingly, ciliate protozoa affect the diversity of ruminal bacteria [42] and end-fermentation products. However, protozoa are not essential for ruminal fermentation and removal of protozoa from the rumen, also called defaunation, does not seem to significantly impact animal health, although feed digestibility might be affected [41]. The potential of defaunation to mitigate CH_4_ emission has been studied [43, 44]. The external surface of protozoa is a site for methanogen attachment (ectosymbionts) or intracellular colonization (endosymbionts), which are attracted by the H_2_ produced in protozoan hydrogenosomes [41]. This symbiosis enhances methanogenesis and favors protozoa as it reduces the levels of H_2_, enabling them to continue the fermentation of monosaccharides left by bacteria. Indeed, the protozoan-associated methanogens are estimated to be responsible for 37% of CH_4_ emission [45], and a meta-analysis including several in vivo studies concluded that a reduction of protozoa concentration was, in most cases, associated with a reduction of CH_4_ emission [46]. Nevertheless, as noted by some authors, defaunation has implications on other metabolic pathways (e.g., fatty acids), which likely justifies a holistic view of the use of this strategy for CH_4_ mitigation [47]. Ionophore additives were suggested for reduction of CH_4_ emission, due to their positive [48] and transient effects [49] on reduction of protozoans and methanogens. However, environmental contamination with still active antibiotics is detrimental for the environment and needs to be considered [50]. Other strategies targeting rumen protozoans include the supplementation of plant metabolites, such as essential oils, which disrupt protozoal membrane, indirectly reducing methanogens [51–53].

### Fungi

More than 90% of fungi sequences isolated in the rumen remain unclassified [54], however, the presence of anaerobic fungi belonging to phylum Neocallimastigomycetes (genera *Neocallimastix*, *Caecomyces*, *Piromyces*, *Anaeromyces*, *Orpinomyces*, and *Cyllamyces*) is acknowledged [55]. Fungi are infrequently found in strained rumen fluid because zoospores attach and colonize (encyst and germinate to produce the fungal thallus) the plant fragments suspended in the rumen natural digesta, being only then released by the sporangia [56]. This may have contributed to a greater unawareness of the importance of their fibrolytic activity. Nonetheless, fungi have enzymes to degrade plant cell wall carbohydrates [55] and are indeed more efficient in degrading lignin than bacteria [57]. In co-culture with methanogens, *Neocallimastix* exhibited high lignocellulose-degrading activity with the production of CH_4_ and acetate [58]. Other studies support the importance of fungi as substrate and electron donors for methanogenesis [59], which makes fungal metabolic pathways an attractive subject for study in the context of the mitigation of CH_4_ emission.

## Ruminal carbohydrate digestion

Plant cell wall polysaccharides are arrangements of glycosidic linkages (e.g., mono-, di-, and oligosaccharides) and noncarbohydrate moieties [60], and might be analytically grouped into cellulose, hemicellulose, and pectin [30]. Cellulose, the most abundant component of cell wall plants, is formed by β-glucose and other hexoses. Whereas, hemicellulose, mainly composed of pentoses with linear xylose chains and variable linkages of arabinose, uronic acids, and galactose, is the second most abundant [61]. Pectin is present in the primary cell wall and has d-galacturonate in its structure [30]. Moreover, starch, a non-structural carbohydrate, is composed of α-glucose, constituting the major carbohydrate storage in plants and an important source of energy for ruminants [62]. Ruminal microorganisms cleave complex glycosidic bonds mainly through glycoside hydrolases. Indeed, the enrichment in those enzymes observed in Bacteroidales, including *Prevotellaceae*, Fibrobacteres, and some Clostridiales [63], affords them a competitive advantage justifying its higher abundance in the rumen.

**Fig. 2:**
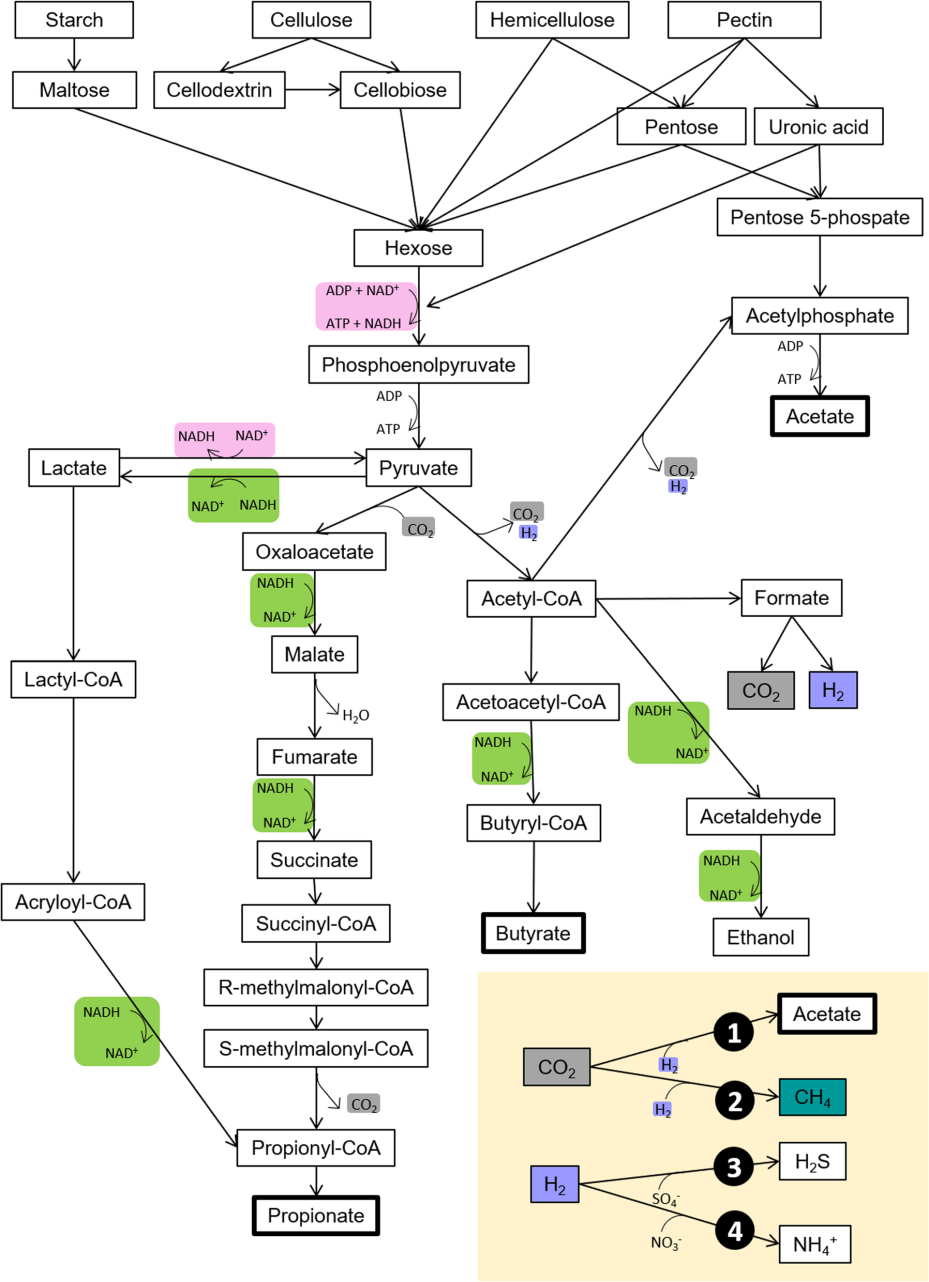
Scheme of rumen carbohydrate fermentation pathways into volatile fatty acids (bold lined boxes) and other intermediate metabolites. The rumen H_2_ sink pathways are displayed: reductive acetogenesis (1), methanogenesis (2), sulfate reducers (3), and nitrate reducers (4)

Chewing and rumination are important for carbohydrate digestion carried out by ruminal microorganisms, as it facilitates the adhesion of bacteria to plants, hydrating and disrupting the protective cuticular layer of plants [29]. After specific adhesion, in which bacterial-substrate linkages and adhesins are developed, the proliferation and colonization of plant tissues are initiated [29]. This is possible because the fermentation of sugars leads to the formation of ATP, the main source of energy for microorganisms [61]. Microbial enzymes degrade the hexoses primarily through the Embden-Meyerhof pathway, originating NADH and pyruvate. Hemicellulose has two pathways for degradation, the transketolase and transaldolase reactions of the pentose cycle or phosphoketolase, with the products originated entering the Embden-Meyerhof pathway [61]. Continuation of the metabolism of the intermediate compound pyruvate is dependent on the oxidation of cofactors (e.g., NADH) through pathways that lead to the formation of lactate, succinate, acetate, propionate, butyrate, ethanol, and valerate [64]. The several reactions that occur in rumen fermentation entail the formation and incorporation of metabolic hydrogen [H]. Formation corresponds to the transfer of electron donors of metabolic intermediates to oxidized intracellular cofactors (highlighted in pink in Fig. 2). Conversely, incorporation corresponds to the transfer from reduced intracellular cofactors to metabolic intermediate electron acceptors (highlighted in green in Fig. 2) [65]. This electron transfer is carried out by hydrogenases and originates H_2_. Furthermore, H_2_ and CO_2_ might also be originated from the conversion of formate in the pyruvate-ferredoxin oxidoreductase [64]. Hydrogen is then transferred between producing species (bacteria, protozoa, and fungi) and hydrogenotrophic microorganisms, mainly methanogens.

## Methanogenesis and CH_4_ emission

Rumen methanogenesis is known to occur by three different pathways: hydrogenotrophic (A), acetoclastic (B), and methylotrophic (C) [20]:

(A) $$4H_{2} + CO_{2} \to CH_{4} + 2H_{2} O$$
  $$4HCOOH \to CH_{4} + 3CO_{2} + 2H_{2} O$$
(B) $$CH_{3} COOH \to CH_{4} + CO_{2}$$
(C) $$4CH_{3} OH \to 3CH_{4} + CO_{2} + 2H_{2} O$$
  $$4CH_{3} - NH_{2} + 2H_{2} O \to 3CH_{4} + CO_{2} + 4NH_{3}$$
  $$2(CH_{3} )_{2} - NH + 2H_{2} O \to 3 CH_{4} + CO_{2} + 2NH_{3}$$
  $$4(CH_{3} )_{3} - N + 6H_{2} O \to 9CH_{4} + 3CO_{2} + 4 NH_{3}$$

Hydrogenotrophic methanogenesis is largely the most frequent, which likely occurs because the energetics involved is more favorable, resulting in slower growth rates and lower cell yields for microorganisms involved in the other methanogenesis pathways [22]. The genus *Methanobrevibacter*, a highly abundant hydrogenotroph, has been correlated with high CH_4_ emissions in steers [66], heifers [67], and dairy cows (more particularly *Mbb. gottschalkii* and *Mbb. ruminantium*) [68]. In turn, *Methanosphaera* (methylotroph) was negatively correlated with CH_4_ emission in heifers [67] and dairy cows [69, 70]. This might be explained by the stoichiometric of the reaction, as one mole of CO_2_ is required to produce one mole of CH_4_ (A), while four moles of methanol are required to produce three moles of CH_4_ (B) in the methylotrophic pathway [69]. Interestingly, the methylotrophic pathway appears to be more significant in young calves compared to mature cows [71]. Even though methylotrophs might have a lower CH_4_ yield, they release NH_3_ to add to the amount already produced by proteolytic bacteria [72]. Importantly, methyl-coenzyme M reductase, which catalyzes the final step of methanogenesis is common across the different methanogenesis pathways [73], which is likely an advantage for strategies intending to target directly methanogens. An example is 3-nitrooxypropanol, a molecule that was shown to inhibit methanogenesis by oxidizing the active site Ni(I) of methyl-coenzyme M reductase [74].

Despite the differences reported in microbial abundances, metagenomic and metatranscriptomic sequencing studies showed that the increased expression of methanogenesis pathway genes explains the increase in CH_4_ emissions, which might itself be regulated by a substrate effect [75]. The diversity of methanogens in the rumen has been correlated to CH_4_ emission. A study using co-abundance analysis of rumen microorganisms of cows reported that low-CH_4_ emitting animals had a more diverse community of methanogens involved in the three methanogenic pathways, compared with high-CH_4_ emitting ones, which had low numbers of hydrogenotrophic methanogenic genera [76]. This shows that methanogen diversity is correlated to CH_4_ emission and highlights the interaction between communities and competition among methanogens for H_2_. Also, low-CH_4_ cow emitters exhibited a more complex microbial network composed of a diverse microbiome, more specifically, bacterial and fungal genera and their genes [76]. Indeed, differences in the microbiome of low- and high-CH_4_ emitters are not limited to methanogens, which is not surprising as the availability of precursors for methanogenesis is dictated by the fermentative microbial consortium. Experimentally, *S. dextrinosolvens* in co-culture enhanced a member of Methanomassiliicoccales and inhibited the activity of a member of the *Mbb. gottschalkii* clade [77]. An in vivo study showed that low-CH_4_ emitting sheep were associated with an elevated abundance of l-lactate dehydrogenase genes, an enrichment of genus *Sharpea* (family *Erysipelotrichiacaea*), and decreased abundance of families *Lachnospiraceae* and *Ruminococcaceae* [78]. These results were confirmed by another study using MAG on microbial sequences collected in high and low CH_4_ emitting sheep, allowing the identification of differential abundance of other genera and microorganisms at the species level [31]. Low-CH_4_ emitting sheep had, in addition to previously mentioned, a high abundance of *Kandleria*, *Fibrobacter*, and *Selenomonas* at a genus level, whereas at a species level, *Fibrobacter succinogenes* and several species of *Bifidobacterium*, *Olsenella*, *Desulfobrivio* were more abundant [31], compared to high emitting sheep. In dairy cows, the abundance of genera *Christensenellaceae*, *Mogibacteriaceae*, S24-7, *Butyrivibrio*, *Shwartzia*, and *Treponema* was associated with low CH_4_ emission in one study [69] and with genus *Eubacterium* in another, in which rumen samples of heifers were analyzed [67]. It is important to consider that effects of the host (e.g., ruminant species), diet as well as methodologies (e.g., sequencing technique and/or 16S rRNA hypervariable region selected) might preclude a direct comparison of microbial composition across studies.

Age and physiological states affect the rumen microbiome composition and thus CH_4_ yields, as shown in a study in heifers, in which the interaction of genera *Prevotella* and *Methanobrevibacter* was associated with the CH_4_ yield, while in older cows, the significant interaction was between *Methanobrevibacter* and *Succinivibrio* [79]. Interestingly though, a study reported that early and late lactation stages were correlated with different levels of CH_4_ emissions not accompanied by changes in the rumen microbiome [80]. In a study with Colombian buffalos, the genus *Prevotella* was associated with low CH_4_ emissions [81]. Despite the differences reported in archaea composition between buffalo and cattle [82], this bacterial group seems relevant for CH_4_ emission of both species. As some authors pointed, the host genotype affects the phenotype of CH_4_ emission, in addition to its microbial community [81]. Indeed, one study indicated that H_2_-producing bacteria explained up to 24% of CH_4_ phenotypic variance, and host genome, 14% [70]. In another study, the CH_4_ emission had a cumulative effect of archaea and bacteria of 13% and 21% of host genetics [83]. Despite the percentage difference, both studies suggest that targeting the rumen microbiome of low-CH_4_ emitting animals (through breeding programs) is possible and likely more effective than not considering the host genome and heritability of the trait.

## Alternative pathways to H_2_ sink

As previously stressed, the concentration of H_2_ determines the CH_4_ produced, while the production of H_2_ is determined by the prevailing pathways of glucose fermentation. The fermentation into butyrate (D) and acetate (E) entails a potential production of one mole of H_2_ (per mole of glucose), whereas propionate (F) entails a net incorporation of one mole of H_2_ (per mole of glucose) [65]. This balance considers the reducing equivalents [2H] produced and incorporated through several reactions (Fig. 2). Microbial cells able to change their fermentation patterns and better adapt to certain conditions are likely more active degrading the available substrates, thus managing to expand [84]. The concentration of H_2_ conditions the fermentation pathway, affecting the free energy change between reactants and products in which the microbial biomass thrives. Summarily, high concentrations of H_2_ favor the productions of propionate, whereas low concentrations of H_2_ favors the production of acetate [11]:

(IV) $$C_{6} H_{12} O_{6} \to 2CH_{3} CH_{2} CH_{2} COO^{ - } + CO_{2} + 2H_{2} + H^{ + }$$
(V) $$C_{6} H_{12} O_{6} + 2H_{2} O \to 2CH_{3} COO^{ - } + 2CO_{2} + 2H^{ + } + 4H_{2}$$
(VI) $$C_{6} H_{12} O_{6} \to CH_{3} CH_{2} COO^{ - } + 2H_{2} O + 2H^{ + }$$

A recent study analyzing MAGs of the microbiota of gastrointestinal ruminants reported that 48% encoded enzymes for fermentative H_2_ production, 1.5% for H_2_-uptake hydrogenases and the methyl-coenzyme M reductase (mcrA genes) related to hydrogenotrophic methanogenesis, while 11% encoded both hydrogenases and the required terminal reductases of alternative methanogenesis pathways. Acetogenesis constituted 3% of MAGs, fumarate 1.9%, and sulfate reduction 0.8% [26]. It has been proposed that the microbial hydrogenases and fermentation pathways are differentially regulated through direct H_2_ sensing by putative sensory [FeFe]-hydrogenases [15]. Indeed, sensory hydrogenases, as well as fermentative and bifurcating hydrogenases, are highly expressed in Clostridiales, Bacteroidales, and Selenomonadales, whereas methanogenic hydrogenases are present, by order of expression, in Methanobacteriales > Methanomassiliicoccales > Methanosarcinales [15]. Moreover, low-CH_4_ emitting sheep were reported to have higher hydrogenase and terminal reductase transcripts from alternative H_2_ uptake pathways, which might even serve as a larger H_2_ sink than methanogenesis, compared to high-CH_4_ emitters [15]. This highlights the preponderant role of the bacterial consortium in determining H_2_ metabolism and the strategies of methanogens to compete and affect H_2_ utilization, and thus CH_4_ emission.

Dietary manipulation of H_2_ production has been attempted to reduce CH_4_ emissions, and it is highly associated with changes in ruminal microbiota. Higher concentrations of H_2_ are associated with high starch content diets, likely because H_2_ release outgrows the capacity of H_2_-consuming microorganisms, leading to an accumulation [85]. Hydrogen accumulation was also associated with diets containing tannin-rich peanut skin provided to beef cattle due to the reduction of H_2_-using microorganisms, including populations of Bacteroidetes phylum, total methanogens, *Methanobrevibacter*, and protozoa, concomitantly reducing methanogenesis [84]. Supplementation of non-fermentative sources, such as Mg, was shown to increase ruminal dissolved H_2_, which affected the microbiota, decreasing the copy number of fungi in goats [86].

In addition to diet effects, host factors and H_2_-producing microorganisms interact, conditioning H_2_ production. Indeed, a study in sheep reported that animals with a smaller rumen volume emitted proportionally less CH_4_, presumably because their higher feed rate passage selected microorganisms able to grow on soluble sugars that can be quickly degraded [78], thus increasing H_2_.

Directly targeting methanogens and potentiating other H_2_-using microorganisms (e.g., propionate pathway, reductive acetogenesis, nitrate, and sulfate reduction) have been proposed as strategies to mitigate CH_4_ emissions. Sulfate-reducing bacteria (e.g., nitrate-reducing propionibacteria, *Wolinella succinogenes*, and *Veillenolla parvula*) can reduce nitrate and nitrocompounds into N_2_O and NH_4_ [87], and although studies [88] have proven it effective in decreasing methanogenesis, concerns over toxicity have hindered its use and motivated further research to develop safer additives (e.g., encapsulated nitrite [89]). Sulfate-reducing bacteria (e.g., *Desulfovibrio*) reduce sulfate into H_2_S, competing with methanogens for H_2_ and reducing methanogenesis if ruminal levels of sulfate increase [90]. Despite thermodynamics and matrix affinity favors sulfate reduction over methanogenesis, as seen in marine sediments [90, 91], the product is highly toxic to the animal.

### Propionate pathway

Ruminal propionate originates mainly from succinate and acrylate pathways. The fermentation of carbohydrate-rich diets with high levels of soluble sugars promotes the proliferation of amylolytic microorganisms such as *S. bovis* [92]*, Lactobacillu*s, and *Bifidobacterium* [93] that reduce pyruvate into lactate. In physiological conditions (no acidosis), microorganisms such as *M. elsdenii* and *Coprococcus catus* produce propionate from lactate via the acrylyl-CoA, using the acrylate pathway [94]. Indeed, both *M. elsdenii* and *C. catus* were correlated to higher feed efficiency and lower CH_4_ emissions in dairy cows [95].

The production of propionate from lactate can also occur with a first oxidation to pyruvate, followed by carboxylation to oxaloacetate, reduction to malate, dehydration to fumarate, reduction to succinate, and a final decarboxylation to propionate (Fig. 2) [96]. This, also called the ‘randomizing’ pathway, might be carried out by microorganisms such as *S. ruminantium* [97] and *Succiniclasticum ruminis* (unable to ferment substrates other than succinate) [98]. According to an in vitro study, the addition of fumarate-reducing bacteria, *Mitsuokella jalaludinii*, lowered methanogen DNA copies and occurrence through competition for H_2_ [99]. Similarly, supplementation with enterococci (*E. faecalis* and *E. faecium*) exhibited fumarate reductase activity, with an increase of propionate and a decrease of CH_4_ [100]. Another in vitro study reported the potential effects of propionic bacteria on ruminal feed degradation by showing a reduction of CH_4_ from 8 to 20% associated with one strain of *Propionibacterium jensenii* and two of *Propionibacterium thoenin* [101]. *Lactiplantibacillus plantarum* was also shown to decrease in vitro CH_4_ production and increased propionate [102]. In turn, an in vivo study showed that, when provided for 4 weeks to lactating primiparous cows fed contrasting high-starch or high-fiber diets, *Propionibacterium freudenreichii*, *Lactiplantibacillus pentosus*, and *Lactobacillus delbrueckii subsp. bulgaricus* did not affect CH_4_ emissions [103]. This highlights the need to further study the in vivo effects of lactic acid bacteria to elucidate any potential correlation with the in vitro positive results. The use of lactic acid bacteria requires finding delivery options that have already been implemented in the global farming system (e.g., silage inoculants and direct-fed microbes), a challenge that must be addressed [104].

Manipulation of fiber content, namely by the replacement of forage fiber by non-forage fiber sources, promoted an expansion of Firmicutes over Bacteroidetes and of *Methanobrevibacter* over *Methanomassiliicoccus*, and a successful shift of H_2_ flow towards the propionate pathway [105]. Furthermore, feed supplementation with saponin (which causes cell rupture and lysis of protozoan) was shown to shift fermentation products, lowering butyrate and increasing propionate [106].

In vivo studies associated other bacteria such as genera *Succinivibrio* (family *Succinivibrionaceae*), *Roseburia*, and *Blautia* (family *Lachnospiraceae*) with the increase of propionate, when testing diets that differed in the corn processing techniques [107]. Furthermore, family *Succinivibrionaceae* were effectively associated with high propionate and low CH_4_ yield [66, 108].

Adding to its potential as a methanogenesis competitor, the increase of propionate production is advantageous for cows’ health and efficiency. The major share (50 – 75%) of propionate produced is absorbed into the portal vein [109]. Propionate is the main precursor of hepatic gluconeogenesis and essential to supply glucose to the mammary gland, thus contributing to support milk production [110]. Furthermore, infusions of propionate led to greater plasma progesterone concentrations post-ovulation, which can affect follicular development and pregnancy rates, and thus improve reproductive efficiency [111].

### Reductive acetogenesis

Acetogenesis has been documented as an alternative to methanogenesis, in which via the acetyl-CoA pathway, two moles of CO_2_ and four moles of H_2_ are incorporated per one mole of acetate produced (G), in a thermodynamically feasible set of reactions.

(G) $$4H_{2} + 2CO_{2} \to CH_{3} COOH + 2H_{2} O$$*.*

Acetogens degrade multiple substrates (e.g., pentoses, hexoses, alcohols, formate, and methyl groups) in addition to H_2_, and in the rumen of cows and sheep, the dominant microorganisms are members of *Lachnospiraceae*, *Clostridiaceae*, and *Ruminococcaceae* families [112, 113]. The threshold value for H_2_ utilization is lower for methanogens than it is for acetogens, which renders reductive acetogenesis a disadvantage. Therefore, increasing H_2_ pressure would remove methanogenesis thermodynamic advantage [114]. An in vitro study reported that supplementation of acetogen *Eubacterium limosum* was able to produce acetate when methanogens were suppressed concomitantly, but a minimal change in CH_4_ production was observed when methanogenesis was not inhibited [115]. Similarly, in vitro supplementation with *E. limosum* and *Proteiniphilum acetatigenes* decreased CH_4_ concentrations, increased acetate, whereas in vivo, *P. acetatigenes* was associated with high milk protein, lower somatic cell counts, and lower decline of milk production over 60 days [116].

## Conclusions

Several in vivo studies employing sequencing techniques have revealed distinct ruminal microbial communities between high- and low-CH_4_ emitting animals. That knowledge has recently been prompted by metagenomics and transcriptomics that allow not only studying the rumen microbial composition but also its function, unraveling genes and pathways involved in the metabolism of H_2_. The goal of successful interventions is likely shifting H_2_ from methanogenesis, exploiting natural processes, without compromising rumen physiology. That depends upon the interaction between microbial communities, including several orders of bacteria, archaea, and eukaryotes that encode and express enzymes [15], thus mediating ruminal fermentation pathways. The propionate pathway competes with methanogenesis, improving efficiency. Using additives including probiotics to promote propionate production showed good results in vitro, yet in vivo studies are still required to confirm its efficacy as well as its suitability for widespread use. Dietary management, namely the use of concentrates and high rich-carbohydrate diets that naturally select microorganisms involved in the propionate pathway make this a more suitable strategy for intensive production systems. However, the carbon footprint of diet production and transportation must be placed in the equation. Reductive acetogenesis is suitable for grazing animals, in which degradation of fibrous diets originates H_2_ that can be utilized by acetogens, though a combined strategy to reduce methanogens is required.

Even though knowledge on the ruminal microbiome has expanded, there are still several genes and microorganisms whose characterization and function remains unknown, particularly protozoan and fungi. Recent works have recovered archaeal and bacterial MAGs from metagenomic data, from which new, previously unnoticed, pathways and networks have been discovered. This indicates that the ruminal microbiome study is a work in progress and may provide us new prospects for finding solutions for lowering livestock enteric CH_4_ emissions, thus addressing the role of cattle in the current climate emergency.

## Data Availability

Not applicable.

## Abbreviations

GHG: Greenhouse gases
MAGs: Metagenome-assembled genomes
VFA: Volatile fatty acids
